# Upfront Surgery vs. Primary Chemoradiation in an Unselected, Bicentric Patient Cohort with Oropharyngeal Squamous Cell Carcinoma—A Matched-Pair Analysis

**DOI:** 10.3390/cancers13215265

**Published:** 2021-10-20

**Authors:** Philipp H. Zimmermann, Marijn Stuut, Nora Wuerdemann, Kathrin Möllenhoff, Malte Suchan, Hans Eckel, Philipp Wolber, Shachi J. Sharma, Fabian Kämmerer, Christine Langer, Claus Wittekindt, Steffen Wagner, Bernd Kremer, Ernst Jan M. Speel, Jens P. Klussmann

**Affiliations:** 1 Department of Otorhinolaryngology, Head and Neck Surgery, Medical Faculty, University of Cologne, Kerpener Strasse 62, 50931 Cologne, Germany; nora.wuerdemann@uk-koeln.de (N.W.); malte.suchan@uk-koeln.de (M.S.); hans.eckel@uk-koeln.de (H.E.); philipp.wolber@uk-koeln.de (P.W.); Shachi.Sharma@uk-koeln.de (S.J.S.); jens.klussmann@uk-koeln.de (J.P.K.); 2 Center for Molecular Medicine Cologne (CMMC), Faculty of Medicine, University and University Hospital Cologne, Robert-Koch-Strasse 21, 50931 Cologne, Germany; 3 Department of Otorhinolaryngology, Head and Neck Surgery, Maastricht University Medical Center, 6229 HX Maastricht, The Netherlands; M.Stuut@mzh.nl; 4 Department of Otorhinolaryngology, Martini Hospital, 9728 NT Groningen, The Netherlands; 5 Department of Ororhinolaryngology, Head and Neck Surgery, Medical Faculty, University of Giessen, Klinikstrasse 33, 35392 Giessen, Germany; fapz@me.com (F.K.); christine.langer@hno.med.uni-giessen.de (C.L.); Steffen.Wagner@hno.med.uni-giessen.de (S.W.); bernd.kremer@mumc.nl (B.K.); 6 Department of Mathematics and Computer Science, Eindhoven University of Technology, 5600 MB Eindhoven, The Netherlands; kathrin.moellenhoff@ruhr-uni-bochum.de; 7 Institute of Medical Statistics and Computational Biology, Faculty of Medicine, University of Cologne, 50931 Cologne, Germany; 8 Department of Otorhinolaryngology, Klinikum Dortmund, Beurhausstr. 40, 44137 Dortmund, Germany; Claus.Wittekindt@klinikumdo.de; 9 Department of Pathology, GROW-School for Oncology and Developmental Biology, Maastricht University Medical Center, P. Debyelaan 25, 6229 HX Maastricht, The Netherlands; ernstjan.speel@mumc.nl

**Keywords:** oropharyngeal squamous cell carcinoma, therapy, upfront surgery, primary chemoradiation, matched-pair analysis, survival

## Abstract

**Simple Summary:**

Oropharyngeal squamous cell carcinoma (OPSCC) is a common malignancy of the upper aerodigestive tract with rising incidence. While surgical and non-surgical approaches are applied in curative treatment, none of these has proven superior to date. In this study, we investigated overall survival in an unselected, bicentric cohort of patients with OPSCC and compared upfront surgery vs. primary chemoradiation treatments. A matched-pair analysis was performed to exclude confounding factors and reduce bias. Our results suggest that regardless of the treatment modality chosen, overall survival rates are comparable in both cohorts. As a consequence, future studies on functional outcome of patients with OPSCC are mandatory to identify the treatment modality most likely resulting in improved quality of life in patients with OPSCC.

**Abstract:**

The two pillars of therapy for oropharyngeal squamous cell carcinoma (OPSCC) are upfront surgery and primary chemoradiotherapy. Substantial regional preferences exist with regard to the selection of treatment. Despite new therapeutic approaches, patient survival remains poor, with an approximate overall survival (OS) rate of 50% at five years. This study was conducted to investigate a potential survival benefit depending on the treatment modality in OPSCC patients. We retrospectively collected data of 853 patients with histologically confirmed OPSCC from the Giessen and Maastricht cancer databases. To identify risk factors affecting survival, a Cox-proportional hazard model was applied to 442 patients with complete data sets. Based on this cohort a matched-pair analysis with 158 patients was performed to compare OS rates of patients treated either with upfront surgery or primary chemoradiation. For the collective cohort, patients treated with upfront surgery had significantly improved OS rates compared to patients treated with primary chemoradiation. In the matched-pair analysis adjusted for patients’ T-, N- and HPV-status as well as risk profile, we observed that both treatment approaches offered equivalent OS rates. Our study emphasizes that treatment recommendations should be made whenever possible on the basis of side-effect profiles caused by the therapeutic approach used. To draw further conclusions, results of the ongoing “best of” (NCT2984410) study are eagerly awaited, investigating the functional outcome after treatment of OPSCC patients.

## 1. Introduction

With about 92.000 new cases and 51.000 deaths annually worldwide oropharyngeal squamous cell carcinoma (OPSCC) is one of the most common cancer of the head and neck region [1,2]. Besides alcohol and tobacco, the risk of developing OPSCC is closely related to persistent infections with high-risk human papillomavirus (HPV), predominantly HPV type 16, with incidence increasing [3]. As OPSCC is a complex disease, it should be managed in specialised centres where treatment decisions are made by a multidisciplinary tumour board after consideration of all patient-specific factors. Either upfront surgery with or without adjuvant (chemo)radiation vs. primary (chemo)radiation with or without salvage neck dissection is being applied in curative treatments. Whereas early stages of OPSCC (cT1/cN0/cM0 and cT2/cN0/cM0 cases) are mainly treated with single-modality approaches such as radiotherapy or surgery with ipsilateral neck dissection [1,4], advanced stages of OPSCC usually receive primary chemoradiotherapy or combined treatment modalities consisting of surgical resection, reconstruction of the oropharynx and concomitant chemoradiotherapy according to risk factors. If both methods are equivalent, the patient’s decision applies [5]. However, which form of therapy is ultimately recommended to the patient varies greatly according to the region. Compared to patients with HPV-negative tumors, those with HPV-related tumors display a significantly better 5-year overall survival (OS; 40–50% vs. 80%) [6]. Due to the significant better prognosis and the distinct biological and clinical characteristics of this disease, HPV-related OPSCC are classified as a separate tumor entity for which specific staging rules have been established [7,8,9]. Attempts to reflect the better prognosis of patients with HPV-related OPSCC in less intensive therapy settings to spare high rates of side effects have not been successful [10,11]. While few improvements have been made in recent years in terms of survival of patients with OPSCC, some progress has been made in terms of reducing treatment invasiveness and adverse event rates. The introduction of intensity-modulated radiotherapy (IMRT), better-tolerated chemotherapy, and less invasive surgery have led to higher tolerability and significantly lower peri-therapeutic morbidity. Up to this point no treatment modality has been identified as being superior and prospective randomized trials, such as the “EORTC 1420-HNCG-ROG—Phase III study assessing the “best of” radiotherapy compared to the “best of” surgery (trans-oral surgery (TOS)) in patients with T1–T2, N0 oropharyngeal carcinoma” (NCT2984410) are ongoing at the moment. To investigate the impact of the two main treatment regimens in patients with OPSCC on survival, we analysed the OS of OPSCC patients depending on treatment modality, T-stage, N-stage and HPV status in a bicentric, retrospective cohort from Germany and the Netherlands using a matched-pair analysis.

## 2. Materials and Methods

**Figure 1:**
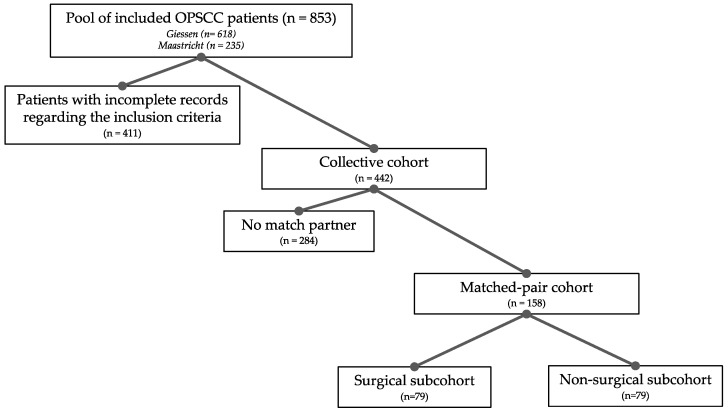
Schematic chart displaying the recruitment process of the cohorts.

Retrospective data were obtained from patient records of 853 patients with OPSCC who were treated with curative intent at the department of otolaryngology, head and neck surgery of the University Hospital Giessen (*n* = 618) or at the department of otolaryngology University Hospital Maastricht (*n* = 235) between the years 2000 and 2015. Diagnosis of OPSCC was based on the International Classification of Diseases for Oncology ((ICD-O); C09, C10). The individual therapy recommendation was made on an interdisciplinary basis in a head and neck tumor board. Whenever possible surgical and non-surgical approaches were offered to each patient. The final decision as to which therapy was chosen, however, depended on regional preferences, which was reflected in an increased rate of the surgical approach in Giessen and an increased rate of radiation therapy in Maastricht. Due to the long-term retrospective nature of our analysis, we cannot present detailed data regarding individual treatment regimens. However, all patients received guideline-compliant therapy at the time of first diagnosis. Furthermore, all surgically treated patients underwent open surgery, as transoral robotic surgery was not yet established at the participating centres at that point. Written informed consent was obtained from all patients. The study was approved by the Ethics Committee of Giessen (AZ 95/15) and Maastricht (METC 11-29-14). Tumour staging and histological grading was assessed according to the 7th edition of the International Union against Cancer (UICC) TNM classification and the WHO criteria for squamous cell carcinomas of the oral mucosa [4,6]. A schematic diagram displaying the workflow of our study is presented in Figure 1.

### 2.1. Inclusion Criteria

For study inclusion, the following criteria had to be met by every individual patient: any T-stage, any N-stage, no distant metastasis (M0), HPV-status of the OPSCC (HPV-positivity defined by p16^INK4a^ immunohistochemistry (IHC) and high-risk HPV-DNA detection by polymerase chain reaction (PCR)) and available data on alcohol and tobacco consumption. Alcohol consumption was assessed using standard units of alcohol (1 SU = 8g alcohol), whereas smoking was assessed using pack years (1 PY = 20 cigarettes a day per year). Patients with regular consumption equal to or greater than 2 SU of alcohol per day and tobacco use of at least one cumulative PY were rated with abuse (“Alcohol Guidelines, Eleventh Report of Session 2010–2012”; UK Parliament House of Commons Science and Technology Committee; 2011; Retrieved September 2021).

### 2.2. Collective Cohort (Complete Data Set) and Multivariate Analysis

Of the initial 853 patients, a total of 442 presented with a complete data set according to the criteria listed above. A Cox proportional hazards model was applied to these 442 patients to investigate the effect of several covariates (mode of treatment, gender, age, TNM, HPV-status, alcohol and tobacco consumption) on survival (Table 1). For the analysis, the assumption of proportionality was checked and met.

### 2.3. Matched-Pair Cohort and Statistical Analysis

From the 442 patients in the collective cohort, 158 patients meeting the matching characteristics could be included in a matched-pair analysis. More precisely, patients treated with upfront surgery were matched against patients receiving primary (chemo-) radiation, according to treatment modality, age (allowed to be in a range of 10 years), TNM-staging, HPV-status and alcohol and tobacco abuse (Table 2). The matching process itself was performed using the SPSS software. We intentionally decided against including the treating centre as a match criterion because we were not aiming for a comparison of two centres but for a comparison of treatment modality. Moreover, this would not have been possible due to inequal cohort sizes. In order to ensure the equality of the matched-pair subcohorts, frequencies were considered for the categorical outcomes and continuous variables were analyzed by running *t*-tests. This enabled the exclusion of confounding factors, and therefore, a survival analysis could be performed with high accuracy and reduced bias, solely regarding treatment differences. As primary endpoint, OS was chosen, defined by the duration from date of diagnosis to date of death. For OS analysis, patients without a date of death were censored at their date of last follow-up. Differences in OS between treatment groups were assessed by calculating Kaplan–Meier estimates and using the log-rank test on equality of survival curves. For evaluation of interdependence of the assessed clinicopathological parameters Fisher’s exact test or Pearson’s Chi-square test were used as appropriate. For all statistical analyses SPSS software (IBM SPSS Statistics Version 25.0. Armonk, NY, USA: IBM Corp.) was used and for all tests a *p*-value < 0.05 was considered as significant.

## 3. Results

### 3.1. Cox Proportional Hazard Model of the Collective Cohort

The multivariate survival analysis of the collective cohort, consisting of 442 patients, suggested superiority of the surgical treatment approach. For patients who underwent upfront surgery, on average a 26% lower risk of death could be observed (HR = 0.745, CI 0.542–1.024, *p* = 0.070), indicating a positive effect compared to the patients treated with primary chemoradiation however, not reaching significance. Mortality of patients with lower T-Stages was significantly reduced (e.g., T-Stage T1 compared to the reference T4b, HR = 0.237, *p* < 0.001) whereas patients with HPV-related tumors had significantly improved survival rates (HR = 0.429, CI 0.285–0.648, *p* < 0.001). The risk factors alcohol and smoking turned out to have a negative, but not significant effect on survival (alcohol: HR = 1.219, CI 0.809–1.836, *p* = 0.344; smoking: HR = 1.497, CI 0.932–2.406, *p* = 0.095; Table 1).

### 3.2. Composition of the Matched-Pair Cohort

In the matched-pair analysis the cohort treated with upfront surgery was composed of 61 (77.2%) patients from the University Hospital Giessen and 18 (22,8%) patients from the University Hospital of Maastricht, whereas the cohort treated with primary chemoradiation was composed of 37 (46.8%) patients from Giessen and 42 (53.2%) patients from Maastricht. Further, in the subgroup treated with upfront surgery, 64 (81%) patients were male and 15 (19%) were female. In the subgroup treated with primary chemoradiation 51 (64.6%) patients were male and 28 (35.4%) were female. The mean age for patients treated with upfront surgery was 59.7 years (standard deviation= 8.1 years). The mean age for patients treated with primary chemoradiation was 59.5 years (standard deviation= 7.5 years). In the cohort treated with upfront surgery, 9 (11.4%) patients presented with Stage I or II disease, 24 (30.4%) patients presented with Stage III disease and 37 (46.8%) patients presented with UICC Stage IV. In the primary chemoradiotherapy cohort, 9 (11.4%) patients presented with Stage I disease, 8 (10.1%) with Stage II disease, 21 (26.6%) with Stage III and 41 (51.9%) patients with Stage IV disease. Surgical and non-surgical cohorts had evenly distributed T-stages with each 17 (21.5%) T1, 28 (35.4%) T2-, 27 (34.2%) T3- and 7 (8.9%) T4-stage cases. Nodal-stage was also evenly distributed between both groups with each group composing of 28 (35.4%) N0-, 13 (16.5%) N1-, 15 (19.0%) N2a-,18 (22.8%) N2b- and 5 (6.3%) N2c-stage cases. High-risk type HPV-infection was present in 17 (21.5%) cases in both therapeutic subcohorts. A total of 70 (88.6%) patients in both the upfront surgery and primary chemoradiation group had a history of alcohol abuse, whereas 69 (87.3%) patients were frequent smokers. It is important to note here the clear difference between the collective cohort and the matched cohort, which is first and foremost that the bias of a more frequent surgical therapy in small tumors with an already better prognosis no longer exists, since the risk factors are now evenly distributed (Table 2).

### 3.3. Impact of Risk-Factors on Overall Survival in the Matched-Pair Cohort

The impact of risk factors on survival in the matched-pair cohort was largely consistent with the impact we calculated from the Cox model in the collective cohort, and was consistent with what was expected from the literature. In the matched-pair cohorts, gender did not have a significant impact on survival although we observed higher survival rates in female patients than in male patients (58.1% female vs. 41.7% male; *p* = 0.066). For the well-known HNSCC risk factors smoking and increased alcohol consumption, we were able to demonstrate a negative influence on OS in case of smoking (*p* < 0.001). Alcohol also demonstrated a trend towards a negative influence on survival, (*p* = 0.080). Higher T-stages were associated with reduced survival rates (*p* < 0.001). Further, the presence of a high-risk HPV-relation in the tumor tissue had a positive influence on OS (38.7% vs. 73.5%, *p* < 0.001). 

### 3.4. Survival Analysis by Therapy Modality in the Matched-Pair Cohort

**Figure 2:**
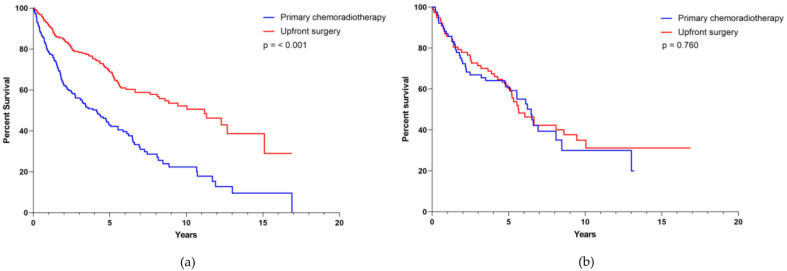
Overall survival (**a**) of the whole, collective cohort (*n* = 442) and (**b**) of the matched-pair cohort (*n* = 158), according to treatment modality applied.

The median survival for the entire collective cohort was 6.2 years (CI 5.027–7.406), whereas for the subgroup treated with upfront surgery in the collective cohort it was 11.2 years (CI 8.362–13.994) versus 4.1 years (CI 2.990–5.284) in the subgroup treated with primary chemoradiation, respectively (Figure 2a, *p* ≤ 0.001). The median survival for the entire matched-pair cohort was 6.1 years (CI 5.230–6.989), whereas for the matched-pair group treated with upfront surgery it was 5.6 years (CI 4.342–6.940) and 6.5 years (CI 5.218–7.724) for the subgroup treated with primary chemoradiation. The two-year estimated OS was 72.4% for the subgroup treated with upfront surgery and 77.9% for the subgroup treated with primary chemoradiation, respectively. Five-year OS was estimated as 61% in patients treated with upfront surgery and 60.3% for patients treated with primary chemoradiotherapy (Figure 2b, *p* = 0.760).

### 3.5. Survival Analysis by Therapy Modality and T-Stage in the Matched-Pair Cohort

**Figure 3:**
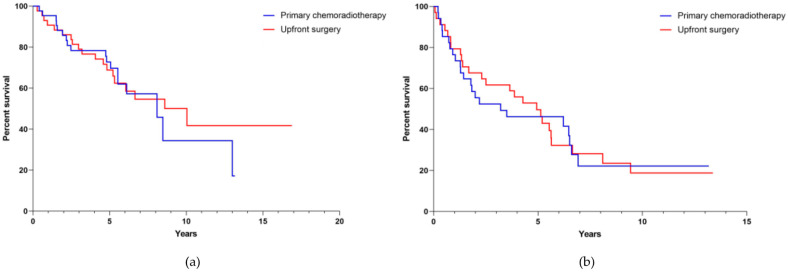
Overall survival (**a**) according to low T-stage (T1–T2,) and (**b**) high T-stage (T3–T4) in the matched-pair cohort (*n* = 158) according to applied therapy. Log-rank tests were stratified for T-stage and are therefore identical for both sub-analyses (*p* = 0.747).

After subdividing the matched groups according to T-stage, patients with T1-2 stage tumors in the subgroup treated with upfront surgery presented with a median survival of 10 years (CI 5.058–15.019, Figure 3a). For patients of the subgroup treated with primary chemoradiation, median survival was 8.1 years (CI 5.558–10.601, Figure 3b). According to advanced tumor stages (T3-4 stage) the median survival in the subgroup with primary surgery was 4.9 years (CI 3.194–6.685) and 3.2 years (CI 0.0–8.389) in the subgroup treated with primary chemoradiation, respectively. There was significant difference between the treatment modalities in patient divided according to T-stage (*p* = 0.747).

### 3.6. Survival Analysis by Therapy Modality and N-Stage in the Matched-Pair Cohort

**Figure 4:**
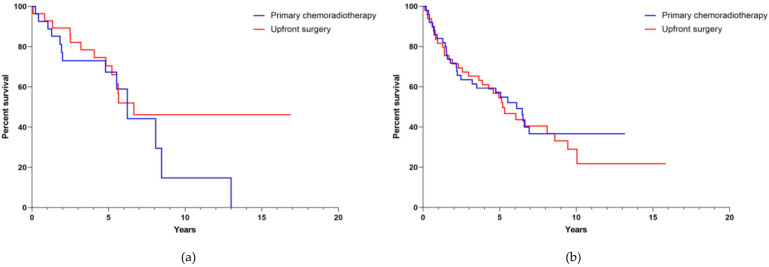
Overall survival (**a**) according to low N-stage (N0) and (**b**) high N-stage (N1-N2c) in the matched-pair cohort (*n* = 158) according to applied therapy. Log-rank tests were stratified for N-stage and are therefore identical for both sub-analyses (*p* = 0.769).

When further subdividing the cohorts according to N-stage, the median survival for patients with N0-stage in the surgical group was 6.7 years (CI could not be computed), whereas for the non-surgical group, it was 6.2 years (CI 4.810–7.623, Figure 4a). For patients with higher N-stages (N1-2c) the median survival in the surgical group was 5.2 years (CI 3.598–6.796) and 6.1 years (CI 4.283–7.937) in the non-surgical group, respectively (Figure 4b). After further subdividing the cohorts into low and high N-stage, there was no significant difference in OS according to treatment modalities (*p* = 0.769).

### 3.7. Survival Analysis by Therapy Modality and HPV-Status in the Matched-Pair Cohort

**Figure 5:**
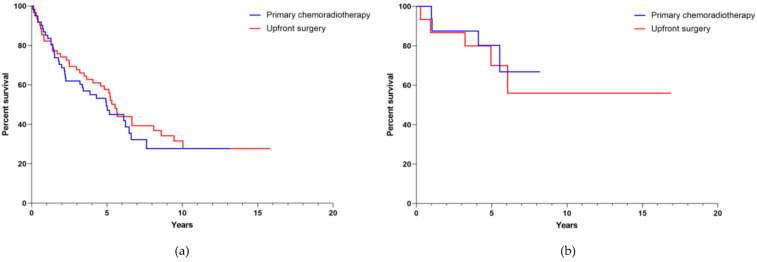
Overall survival (**a**) displaying HPV-negative and (**b**) HPV-related cases in the matched-pair cohort (*n* = 158) according to therapy modality applied. Log-rank tests were stratified for HPV-status and are therefore identical for both sub-analyses (*p* = 0.631).

Subdivision of the cohort according to HPV-status demonstrated a median survival of 4.96 years (CI 3.324–6.594) for non-HPV-related OPSCC patients treated with primary chemoradiation. The median survival in this subgroup treated with upfront surgery was 5.54 years (CI 4.947–6.124, Figure 5a). For HPV-related OPSCC patients the median survival could not be reported as less than 50% of the patients died in the observational period. Further, we were not able to find a significant difference in OS according to treatment modalities when focusing on HPV-status (*p* = 0.631; Figure 5b).

## 4. Discussion

We obtained data from a representative number of OPSCC patients which were either treated with upfront surgery or primary chemoradiation. Two oncological centers at university hospital sites from Germany and the Netherlands working independently of each other and differing in their therapeutic focus, contributed a total of 853 patients with histologically confirmed and newly diagnosed OPSCC. After the initial data set was sorted, a collective cohort of 442 patients was formed with complete data on the known risk profile for OPSCC. For the formation of the collective cohort, high rates of drop outs were due to the fact that patient records from the years 2000–2015 were not always complete and therefore a risk profile could not be established. We first performed a multivariate analysis of survival in the collective cohort to investigate whether the therapeutic approach alone and in correlation with TNM-stage and risk factors as smoking and alcohol had an impact on survival. Kaplan–Meier analysis revealed that patients treated with upfront surgery had a significantly better survival than patients treated with primary chemoradiation (Figure 2a, *p* ≤ 0.001). This observation is in high contrast to the current understanding that, for the treatment of any OPSCC, surgical and non-surgical therapeutic approaches do not differ in terms of survival [12,13,14]. We, therefore, assumed that the observed effect is due to a bias which is most likely caused by the fact the patients with a lower UICC-stage are more likely to receive upfront surgery whereas patients with more advanced stages are predominantly treated with primary chemoradiotherapy. In order to outrun therapy bias and the diverse risk profiles of OPSCC patients, we carried out a matched-pair analysis [15,16]. Those types of analyses have the advantage that the observed cohorts are very similar on the basis of the defined characteristics and thus effects related to co-determining factors are minimized. Therefore, a high level of evidence without actually performing a randomized trial is achieved [12,14,17,18]. Due to the rigid pre-selection, 158 of the initial 853 patients remained for matched-pair analysis, a number that, in the light of OPSCC prevalence, still seems representative. The high dropout rate for the formation of the matched-pair cohorts can be explained by the fact that no match partner was available.

We show that treatment modality had no influence on OS (Figure 2, Figure 3, Figure 4 and Figure 5) and that the median survival of OPSCC patients, regardless of which therapy they received, was approximately 6 years. After matching two patients who differed only in treatment modality, we did not detect a difference in survival in either the group with smaller tumors (T1-2, *n* = 90) or the group with larger tumors (T3-4, *n* = 68). Similarly, when analyzing survival according to lymph node involvement (N0, *n* = 56 vs. N1-N2c, *n* = 102) as well as HPV-status (positive, *n* = 34 vs. negative, *n* = 124) no therapy regimen superior to the other was identified. We believe that the dramatic changes in survival between the collective cohort and the matched cohort support the assumption of a good match process. Since there were 242 cases with T1-2 tumor in the collective cohort and the majority of these cases originated from the Giessen center, most of these cases were treated surgically. This excess of surgically treated cases combined with the known better prognosis of smaller tumors may have resulted in better survival of surgically treated cases in the survival analysis of the collective cohort. However, once this excess was eliminated by matching, there was a comparable oncologic outcome between surgically and non-surgically treated cases, even for smaller tumors. There also appeared to be a trend toward improved survival in patients treated non-surgically, as their overall survival increased when outliers without matching partners were excluded.

In the arm of the study treated with upfront surgery, we did not further differentiate between patients who received surgery alone and those who also received adjuvant treatment. Whether advanced cases treated with surgery and adjuvant therapy benefit primarily from the therapeutic effect of the surgical resection of the tumor and neck dissection, or whether the actual effect on survival only arises from the adjuvant radio (chemo)therapy, is indeterminable from our evaluation (Figure 3).

The initial characteristics of having a lower UICC stage, a lower frequency of alcohol and tobacco consumption as well as a confirmed high-risk HPV-relation of the tumor had a clear positive influence on prognosis of patient in our matched-pair cohort (Table 1, Figure 3, Figure 4 and Figure 5). This is consistent with the data observed by others [19,20,21]. Here, it is worth noting that studies investigating the survival of OPSCC patients which analyzed large national datasets retrospectively already exist. Despite this, the aim of our study was to compare survival data of individually matched patients from two specialized treatment centers providing a different therapeutic focus. In light of OPSCC prevalence, the multiple individual matching criteria, and the long observation period, an initial cohort totaling 853 patients and a later matched cohort of 158 patients is reasonably substantial.

A noteworthy weakness of our study is the retrospective analysis of clinical data. Due to the design of a long-term retrospective analysis, we cannot provide more detailed information on the surgical or radiotherapeutic therapy approach. Neither are we able to present any data on the quality of life or the ECOG status of the patients before and after therapy due to incomplete data. In addition, for the sake of a larger cohort, we did not carry out further sub-analyses according to, e.g., HPV-status as cohort size would have lacked representative size. Though both centers offered high-quality therapy for OPSCC, a certain bias due to the respective therapeutic preference and the resulting increased experience cannot be excluded with certainty. In further studies, the parameter of disease-free survival after initial curative therapy should be addressed in more detail, as it was not possible to determine from our retrospective dataset.

In conclusion, we observed that a therapeutic concept for oropharyngeal squamous cell carcinoma building primarily on upfront surgery or primary chemoradiotherapy offers equal chances of survival to the patients as no treatment modality has been identified as superior by our matched-pair analysis. Although our result indicates that two identical patients have the same chances of survival with both treatment modalities, this does not mean that both treatment modalities are equally suitable for them. In the individual patient, variables exist, that speak for or against one of the treatment modalities. The decision as to which therapy is most suitable has to be made on a case-by-case basis. It is therefore of great importance that both therapies are considered to be of equal value and that corresponding therapy capacities are available in the treating centers in order to have an appropriate concept for each individual patient. Both centers participating in this study were able to offer treatments with either primary chemoradiation or primary surgery in accordance with a high and guideline-based standard. Nevertheless, further prospective studies with lager cohorts are necessary to draw final conclusions on the validity of the therapeutic approaches in OPSCC patients and according to HPV-status. Another emerging change is that an increasing number of young patients with HPV-related OPSCC survive the disease suffering from long-term side effects of therapy. Efforts are mandatory to investigate which therapy modality has the least side effects and in parallel delivers the best functional result. First results of the *ORATOR*-study have demonstrated that patients with low UICC-stage of OPSCC (T1-2, N0-2) displayed a slightly improved swallowing function one year after radiotherapy compared to surgery [22]. Results of the ongoing “best of” (NCT2984410) study are eagerly awaited to shed further light on the functional outcome of patients with OPSCC in accordance to applied therapy concepts.

Our results suggest that regardless of the treatment modality chosen, overall survival rates are comparable in both cohorts. Future studies to focus on functional outcome of patients with OPSCC are mandatory at this point to identify the treatment modality most likely resulting in improved quality of life in this cohort.

Finally, we would like to provide the reader with a list of important points that should be considered pre-therapeutically. After evaluating the basic factors such as tumor location, staging, grading and HPV status, as well as addressing psychosocial factors and life circumstances, the following modality-specific points should be considered when planning a therapy together with the patient. However, we do not claim to offer a comprehensive list of all advantages and disadvantages of surgery and radiochemotherapy in OPSCC, but rather to highlight a few, in our view, substantial points and to offer the reader a mental orientation in the conversation with the patient (Table 3).

## Figures and Tables

**Table 1:** Results of the Cox-proportional-hazard model of the collective cohort.

Variables (*n*)	HR	95% CI	*p*-Value
Treatment			
Primary Chemoradiation (*n* = 208)	1 (Ref)		
Upfront Surgery (*n* = 234)	0.745	0.542–1.024	0.070
Gender			
Female (*n* = 108)	1 (Ref)		
Male (*n* = 334)	1.096	0.786–1.529	0.589
Age at diagnosis (years)	1.027	1.010–1.043	0.001
T-stage			
T1 (*n* = 105)	0.237	0.131–0.430	< 0.001
T2 (*n* = 137)	0.338	0.203–0.564	< 0.001
T3 (*n* = 98)	0.544	0.341–0.871	0.011
T4a (*n* = 68)	0.576	0.350–0.950	0.031
T4b (*n* = 34)	1 (Ref)		
N-Stage			
N0 (*n* = 134)	0.946	0.422–2.125	0.992
N1 (*n* = 64)	0.797	0.342–1.858	0.693
N2a (*n* = 97)	1.349	0.588–3.092	0.385
N2b (*n* = 95)	1.029	0.461–2.297	0.779
N2c (*n* = 42)	1.249	0.541–2.884	0.566
N3 (*n* = 10)	1 (Ref)		
HPV-Relation			
No (*n* = 330)	1 (Ref)		
Yes (*n* = 112)	0.429	0.285–0.648	<0.001
Alcohol			
No (*n* = 81)	1 (Ref)		
Yes (≥1SU/day) (*n* = 361)	1.219	0.809–1.836	0.344
Smoking			
No (*n* = 78)	1 (Ref)		
Yes (≥1PY) (*n* = 364)	1.497	0.932–2.406	0.095

HR = hazard ratio; CI = confidence interval; SU = standard unit; PY = pack year; covariates with a significant effect on survival are marked in bold.

**Table 2:** Characteristics of the matched-pair cohort according to therapy concept applied.

**Characteristics**	**Surgery**	**Non-Surgery**
Patients (*n*)	79	79
Age (years, mean)	59.5	59.7
Gender (*n*)		
Male	64 (81%)	51 (64.6%)
Female	15 (19%)	28 (35.4%)
T Stage (*n*)		
T1	17 (21.5%)	17 (21.5%)
T2	28 (35.4%)	28 (35.4%)
T3	27 (34.2%)	27 (34.2%)
T4a	7 (8.9%)	7 (8.9%)
N Stage (*n*)		
N0	28 (35.4%)	28 (35.4%)
N1	13 (16.5%)	13 (16.5%)
N2a	15 (19.0%)	15 (19.0%)
N2b	18 (22.8%)	18 (22.8%)
N2c	5 (6.3%)	5 (6.3%)
Alcohol (>1 SU, *n*)		
Yes	70 (88.6%)	70 (88.6%)
No	9 (11.4%)	9 (11.4%)
Smoking (>1 PY, *n*)		
Yes	69 (87.3%)	69 (87.3%)
No	10 (12.7%)	10 (12.7%)
HPV (Type 16, *n*)		
Yes	17 (21.5%)	17 (21.5%)
No	62 (78.5%)	62 (78.5%)

SU = standard unit; PY = pack year.

**Table 3:** Presentation of some, in the view of the participating centers, significant advantages and disadvantages of surgery and radiochemotherapy in OPSCC.

**(Primary-) Radiochemotherapy**
Advantage Typically good tumor response (especially in young and healthy patients with positive HPV status) Possibility to modify the protocol (e.g., fractionation and concomitant chemotherapy) Independence from surgical resectability and need for general anesthesia Lower risk of acute complications typically associated with surgery, such as embolism, infection, or pneumonia Disadvantage Longer duration of therapy (approximately 5–7 weeks, depending on radiation protocol) Usually concomitant administration of chemotherapy Damage to surrounding tissues (e.g., skin, mucosa, vessels, bones) Possible necessary interventions to maintain bodily functions (tracheostomy, gastrostomy)
**(Primary-) Surgery**
Advantage Possibility of rapid tumor control with en bloc resection and removal of affected lymph nodes Lower adjuvant radiation dose necessary, possibly without chemotherapy More radiation reserves for the possible case of local recurrence Lower risk of radiation-associated side effects Disadvantage Need for general anesthesia with associated risks Possible necessary interventions to maintain bodily functions ( tracheostomy, gastrostomy) Possible need for reconstruction with free flaps to achieve good functional and aesthetic results Risk of prolonged hospitalization due to perioperative complications (e.g., embolism, infection, or pneumonia)

## Data Availability

The data presented in this study are available on request from the corresponding author. The data are not publicly available because it is an on-site dataset consisting of patient data from the treating centers.
